# Sequencing of Coastal Lagoon Samples from the Piñones Lagoon, Puerto Rico, Reveals Important Role of Bacterial Sulfur Metabolism in the Lagoon Ecosystem

**DOI:** 10.1128/MRA.00172-21

**Published:** 2021-04-29

**Authors:** Fabiola A. Aviles, John A. Kyndt

**Affiliations:** a College of Science and Technology, Bellevue University, Bellevue, Nebraska, USA; University of Delaware

## Abstract

This initial microbial analysis of the Piñones Lagoon shows a high representation of sulfur-oxidizing *Sulfurimonas* and sulfur-reducing *Sulfurospirillum* bacteria. These species are likely responsible for maintaining sulfur homeostasis and prevent the buildup of toxic sulfur components, but may contribute to nitrogen buildup, in the mangrove ecosystem.

## Abstract (precis)

This initial microbial analysis of the Piñones lagoon shows a high representation of sulfur-oxidizing *Sulfurimonas* and sulfur-reducing *Sulfurospirillum* bacteria. These species are likely responsible for maintaining sulfur homeostasis and prevent the buildup of toxic sulfur components, but may contribute to nitrogen buildup, in the mangrove ecosystem.

## ANNOUNCEMENT

The Piñones Lagoon in Loíza, Puerto Rico, is part of the San Juan Bay Estuary and is known for its bioluminescent properties and marine diversity (1, 2). The Department of Natural and Environmental Resources in Puerto Rico has prohibited cutting mangrove to preserve the natural habitat in this reserve. The lagoon has been impacted since 2016 by sedimentation problems related to the mangrove growing in the canals that joined the Piñones Lagoon to the Torrecillas Lagoon (1). Mangrove ecosystems have been proposed for wastewater treatment (3, 4), although recent reports on the San Juan Estuary indicate that excessive nitrogen buildup has larger ecological consequences (2, 5). Understanding the bacterial composition provides insight into the environmental impact that the bacterial microbiome has on the local ecosystem.

**FIG 1:**
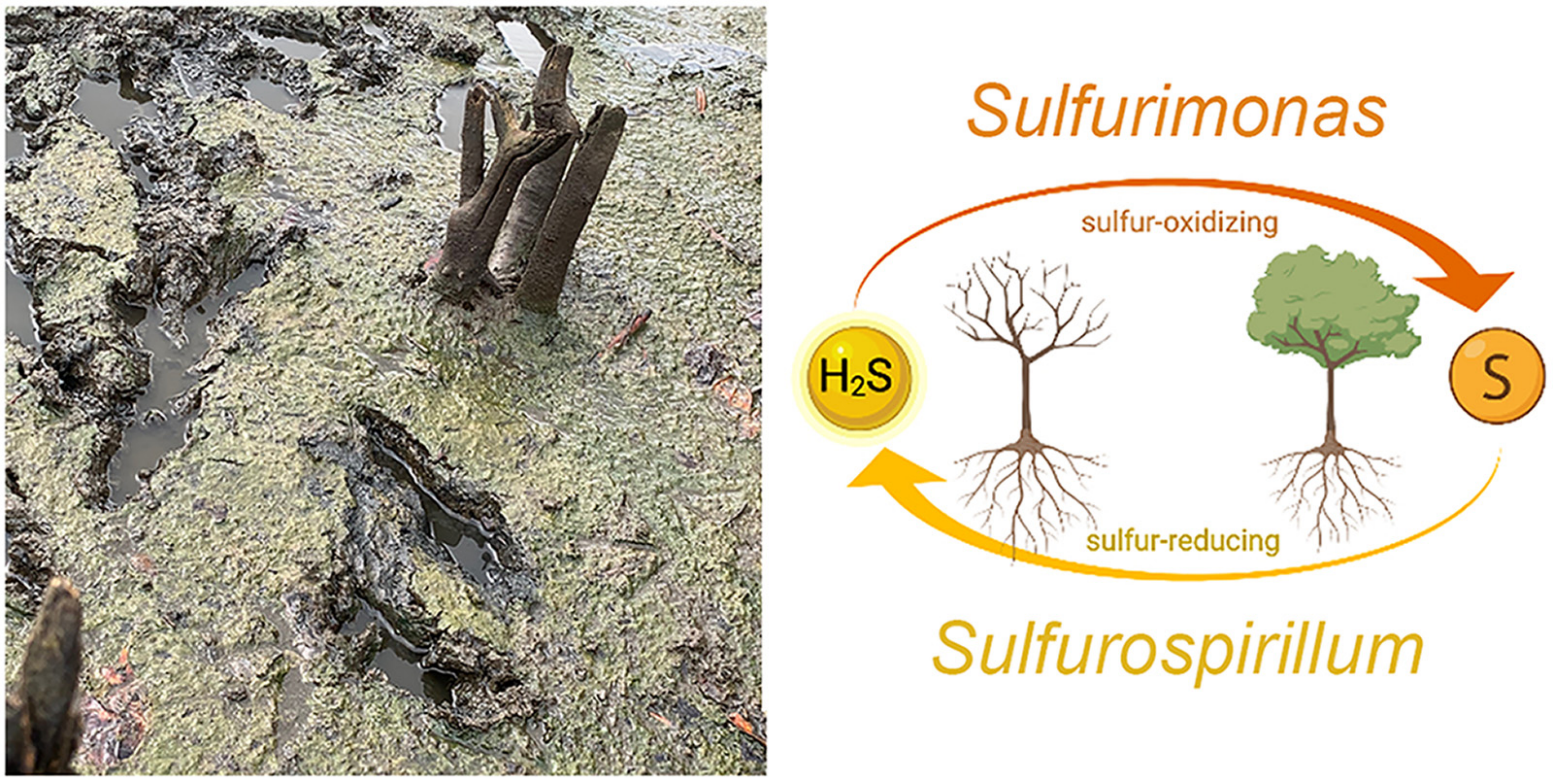
Sampling site and schematic overview of the proposed sulfur cycle, based on the abundance of sulfur-oxidizing *Sulfurimonas* species and sulfur-reducing *Sulfurospirillum* species in the Piñones Lagoon microbiome. *Sulfurimonas* oxidizes the toxic sulfide produced by *Sulfurospirillum* to inorganic sulfate, which is utilized by the mangroves.

A water sample (from the top 10 cm of the water column) was collected in triplicate (isolates FABPR1, FABPR2, and FABPR3) from the Piñones Lagoon (latitude, 18.4434427; longitude,−65.956203) (Fig. 1) in March 2020. Samples were collected in sterile 15-ml tubes, stored in a cooler with ice packs for ∼2 h, and transferred to 4°C. The water phase (top 10 ml) of each sample was transferred by pipetting to a new sterile tube, leaving most of the sediment behind. DNA extraction was performed on the transferred water phase using the PureLink microbiome DNA purification kit (Thermo Fisher). Nanodrop analysis showed an *A*_260_/*A*_280_ of 1.60 to 1.70. A 16S rRNA amplicon sequencing library was prepared following the 16S metagenomic sequencing library protocol (Illumina) (6). The 16S amplicon primers targeting the V3 and V4 regions were as described in the protocol and were synthesized by Sigma (7). The samples were sequenced using a 1.8-pM library with an Illumina MiniSeq instrument. Paired-end (2 × 150 bp) sequencing generated 1,002,554 reads (FABPR1), 1,213,886 reads (FABPR2), and 335,002 reads (FABPR3). The primer sequences were removed, and reads with a low quality score (average score, < 20) were filtered out using the FASTQ toolkit (version 2.2.0) within BaseSpace (Illumina). The 16S Metagenomics app (version 1.0.1) within BaseSpace version 1.0.1 was used to perform a taxonomic classification, which uses an Illumina-curated taxonomic database, RefSeq RDP 16S version 3 (8), and the RDP naive Bayes taxonomic classification algorithm was used with an accuracy of >98.2% at the species level (9). Default parameters were used for all software.

Table 1 provides an overview of the most abundant operational taxonomic units (OTUs) at the genus level. The triplicate samples gave nearly identical results and are dominated by genera that play a role in environmental sulfur metabolism. Sulfur is an important element in the metabolism of salt marshes and subtidal, coastal marine sediments because of its role as an electron acceptor, carrier, and donor (10, 11), and sulfur redox cycling is often found to be important for syntrophic relationships in bacteria (12–14). *Sulfurimonas* and *Sulfurospirillum* species have been identified in distinct environments such as hydrothermal deep-sea vents, marine sediment, and terrestrial habitats (11, 15–17). *Sulfurimonas* species are sulfur-oxidizing bacteria, while *Sulfurospirillum* species are sulfur-reducing, nitrogen-fixing bacteria (Fig. 1). It has recently been shown that *Sulfurimonas* species play an important role in other mangrove environments for the oxidation of the toxic sulfide produced by sulfur-reducing bacteria such as *Sulfurospirillum* species. These species likely play a similar role here for detoxification and maintenance of homeostasis in the lagoon ecosystem, although overgrowth of N-fixing, sulfur-reducing bacteria may contribute to buildup of nitrogen in these estuaries (2, 5).

**TABLE 1:** Overview of the bacterial diversity at the genus level, based on 16S rRNA gene amplicon analysis^*a*^

Classification	Total reads (%)	SD
*Sulfurimonas*	54.79	1.00
*Sulfurospirillum*	7.68	0.32
*Fusibacter*	1.80	0.04
*Nitrincola*	1.71	0.08
*Mycoplasma*	1.08	0.01
*Hydrogenimonas*	1.01	0.08
Unclassified	7.73	0.31

a The values for the triplicate samples are provided as calculated averages (standard deviation [SD]) of the percentage of total reads. Only genera with an abundance of >1% of the reads are represented.

### Data availability.

The 16S rRNA gene amplicon data sets have been deposited at DDBJ/ENA/GenBank under BioProject number PRJNA701415 and can be accessed under the SRA accession numbers SRR13687041 (FABPR1), SRR13691807 (FABPR2), and SRR13691798.
